# The Impact of the COVID-19 Pandemic on Osteoporosis Diagnosis and Treatment in Iran: A National Study

**DOI:** 10.5812/ijem-165816

**Published:** 2025-10-31

**Authors:** Sepideh Hajivalizadeh, Mahnaz Sanjari, Kazem Khalagi, Mohammad Javad Mansourzadeh, Saeed Shahsavari, Amirhossein Aghakhani, Mohammad Effatpanah, Zahra Shahali, Fatemeh Hajivalizadeh, Pardis Zarepour, Parastoo Montazerlotf, Elahe Hesari, Noushin Fahimfar, Afshin Ostovar

**Affiliations:** 1 Osteoporosis Research Center, Endocrinology and Metabolism Clinical Sciences Institute, Tehran University of Medical Sciences, Tehran, Iran; 2 Obesity and Eating Habits Research Center, Endocrinology and Metabolism Clinical Sciences Institute, Tehran University of Medical Sciences, Tehran, Iran; 3 Prevention of Metabolic Disorders Research Center, Research Institute for Endocrine Sciences, Shahid Beheshti University of Medical Sciences, Tehran, Iran; 4 Department of Biostatistics and Epidemiology, School of Health, Alborz University of Medical Sciences, Karaj, Iran; 5 Department of Pediatric, Imam Khomeini Hospital, School of Medicine, Tehran University of Medical Sciences, Tehran, Iran; 6 National Center for Health Insurance Research, Tehran, Iran; 7 Department of Public Health, Center for Non-communicable Disease Control and Prevention, Ministry of Health and Medical Education, Tehran, Iran; 8 Department of Epidemiology and Biostatistics, School of Public Health, Tehran University of Medical Sciences, Tehran, Iran; 9 Epidemiology and Biostatistics Unit, Endocrinology and Metabolism Research Institute, Tehran University of Medical Sciences, Tehran, Iran; 10 Non-communicable Diseases Research Center, Endocrinology and Metabolism Population Sciences Institute, Tehran University of Medical Sciences, Tehran, Iran; 11 Endocrinology and Metabolism Research Center, Endocrinology and Metabolism Clinical Sciences Institute, Tehran University of Medical Sciences, Tehran, Iran

**Keywords:** Alendronate, Bone Mineral Density (BMD), Calcitonin, Diagnosis, Disease Management, Medication, Osteoporosis, SARS-CoV-2, Treatment

## Abstract

**Background:**

The emergence of the COVID-19 pandemic disrupted the management of non-communicable diseases, including osteoporosis.

**Objectives:**

This study aims to investigate the impact of the COVID-19 pandemic on osteoporosis diagnosis and treatment in Iran.

**Methods:**

This cross-sectional study evaluated changes in the number of prescriptions for osteoporosis diagnosis and treatment before and during the COVID-19 pandemic from all available and complete data. Data on the number of prescriptions for bone mineral density (BMD) tests, osteoporosis-related lab tests (serum vitamin D level and serum calcium level), and medications (alendronate and calcitonin) were obtained from the Iran Health Insurance Organization (IHIO) dataset from March 2019 to March 2022. Additionally, the University of Sheffield provided the number of fracture risk assessment tool (FRAX^®^) used with the internet protocol (IP) addresses of Iran from 2019 to 2023. Statistical analysis was performed using the Mann-Whitney test and the interrupted time series analysis utilizing STATA. This research was carried out with the support of the Endocrinology and Metabolism Research Institute, Tehran University of Medical Sciences, Tehran, Iran, under project code 1400-02-103-1177.

**Results:**

A total number of 4,901,027 prescriptions composed of 62,718 prescriptions for BMD tests, 2,862,871 prescriptions for serum vitamin D level tests, and 1,952,600 prescriptions for serum calcium level tests regarding diagnosis, and a total number of 446,791 prescriptions, including 388,519 alendronate and 58,272 calcitonin prescriptions regarding treatment, were evaluated. A statistically significant decrease in the number of prescriptions for BMD test and FRAX^®^ usage, with regression coefficients of -2583.3 and -410.5, respectively, was demonstrated. A significant and nonsignificant increase, with regression coefficients of 71579.4 and 814.1 regarding the number of prescriptions for serum vitamin D and serum calcium tests, respectively, was highlighted. A statistically significant decrease in the number of prescriptions for alendronate and calcitonin, with regression coefficients of -9592.0 and -2272.3, respectively, was noted.

**Conclusions:**

The present study demonstrated that the COVID-19 pandemic negatively affected the diagnosis and treatment of osteoporosis. However, it was limited by the lack of data on prescriptions for other osteoporosis medications and diagnostic tests, the lack of access to the data on prescriptions for people whose costs are not covered by the IHIO, and the restricted data access regarding the presented data. The findings are based on prescription-level data in Iran and primarily reflect practice within this healthcare context; therefore, caution is required when generalizing to settings with different healthcare systems or population structures. Further studies are needed to assess this impact and to help prevent management disruption in future health crises.

## 1. Background

The emergence of the 2019 novel coronavirus (COVID-19) pandemic necessitated all specialties to contribute to COVID-19-related medical care and affected the management of other health problems (1). Many healthcare services, including elective surgeries and outpatient visits, were postponed in many countries because of the COVID-19 pandemic. Decreasing infection risk and hospital burden were two reasons among many (2). Following the onset of the COVID-19 pandemic, concerns related to this health crisis led to delays in managing other diseases by reducing patients' access to health services, delaying treatment, and altering clinic schedules (3).

Non-communicable diseases (NCDs), including osteoporosis, were among the most affected health problems. They were influenced by disruption in prevention and control (4). Osteoporosis is a prevalent chronic disorder affecting more than 200 million individuals globally, with a prevalence of 38% in women and 25% in men in the Iranian population (5). It is the most prevalent metabolic bone disease, having led to 150,000 osteoporotic fractures, caused 3,554 deaths, and incurred an estimated economic burden of US$393.24 million in Iran in 2020 (6).

Based on studies, devoting the workforce previously dedicated to outpatient services to inpatient ones because of the pandemic reduced so much of these services, resulting in less effort for osteoporosis diagnosis and treatment (1). The pandemic caused a reduction in general practitioner visits, specialist practitioner visits, the number of performed bone mineral density (BMD) tests, vitamin D tests, and treatment initiations (7). During the pandemic, diagnostic imaging had been given priority depending on clinical urgency. In several centers, dual X-ray absorptiometry (DXA) services were not considered a priority or were temporarily suspended. In centers where these services have been able to continue operating, the throughput has significantly decreased due to strict infection control strategies and social distancing. Furthermore, patients at an increased risk of infection and aged individuals who were especially prone to fragility fractures linked to osteoporosis were hesitant to visit hospitals due to their fear of COVID-19 transmission (8). Therefore, the preexisting challenges in osteoporosis management were exacerbated by the emergence of the COVID-19 pandemic (8).

## 2. Objectives

This study aims to highlight the effect of the COVID-19 pandemic on osteoporosis management by comparing the diagnosis and treatment of osteoporosis before and during the pandemic, specifically from 2019 to 2022, in Iran.

## 3. Methods

### 3.1. Study Design and Data Source

This was a cross-sectional study utilizing the data of the Iran Health Insurance Organization (IHIO), also known as “Bime Salamat,” covering all 31 provinces of Iran. Iran Health Insurance Organization is a leading government-operated insurance company that covers over 40 million Iranians, offering accessible essential insurance services at an affordable rate (9). Iran Health Insurance Organization covers the health-related expenses of about 45% of the Iranian population and is one of the leading national health insurance services in Iran. The data were accessed for research purposes from IHIO on December 26, 2022.

All available data on related tests and medications were evaluated to investigate the effect of the pandemic on the diagnosis and treatment of osteoporosis. Regarding diagnosis, the insurance provided the number of prescriptions for BMD testing, serum vitamin D level tests, and serum calcium level tests. Furthermore, the fracture risk assessment tool (FRAX^®^) is an algorithm (https://FRAX.shef.ac.uk/FRAX/) developed by the World Health Organization Collaborating Centre for Metabolic Bone Diseases. It is a computer-based tool now integrated into numerous clinical guidelines (10). We communicated with the University of Sheffield via the official email address of the Osteoporosis Research Center. We formally requested access to data on the usage of FRAX^®^ with all internet protocol (IP) addresses in Iran. The data were accessed for research purposes from the University of Sheffield on June 12, 2023. Regarding treatment, prescriptions for all forms and dosages of alendronate and calcitonin were obtained from IHIO and investigated.

Since the COVID-19 pandemic was announced in Iran in February 2020, data were supplied as the most available for this period to compare the management of osteoporosis before and during the COVID-19 pandemic. The most available data regarding prescriptions for BMD tests, alendronate, and calcitonin were between March 2019 and March 2022, and prescriptions for serum vitamin D tests and serum calcium tests were from August 2019 to March 2022. Concerning FRAX^®^, data from January 2019 to May 2023 were evaluated. Electronic prescribing systems, or e-prescribing, have been officially required in Iran since December 22, 2021 (11). Hence, we merged the number of prescriptions for both forms, and the reported numbers comprised electronic and paper forms.

Prescriptions for osteoporosis tests, including BMD tests, serum vitamin D tests, serum calcium tests, and FRAX^®^ usage, were assessed separately from all available data. For BMD tests, 10 months before the pandemic and 26 months after it were evaluated. Regarding serum vitamin D tests and serum calcium tests, data for six months before the initiation of the pandemic and 26 months after the beginning of it were assessed. For FRAX^®^, the assessment included 15 months before and 38 months after the pandemic.

### 3.2. Statistical Analysis

The data obtained from the IHIO dataset underwent cleaning and preparation for statistical analysis. We gathered and merged our data from two different sources, paper prescriptions and electronic prescriptions from each province of Iran (n = 6,401,137). Any identical prescription records for the same patient on the same day were removed to prevent double-counting (n = 147,244). Prescriptions that were incorrectly coded or did not pertain to the specific osteoporosis-related diagnostics and treatments under investigation were filtered out (n = 1,352,866). These data used a variable called “service code” indicating the type of service the patients received. We used these codes to create our outcome variables. Then the data was summarized into aggregated data to count the number of prescriptions by months.

Using the Mann-Whitney test, the median number of drugs or diagnostic tests prescribed per month was calculated and compared between the pre-COVID-19 pandemic and during the pandemic periods. Interrupted time series analysis was employed to assess the time trends in the quantity of prescribed medications or diagnostic tests across the periods, as mentioned earlier. This analysis facilitated the estimation of the time trend in drugs or diagnostic test prescriptions before and during the pandemic, as well as the associated changes and shifts in prescription levels at the onset of the pandemic. We also conducted a cross-correlation analysis to determine if the peaks in reported new COVID-19 cases were correlated with changes in the number of osteoporosis-related prescriptions, including a time lag to account for delayed effects. The statistical analyses were conducted using STATA 15.0 statistical software (Stata LLC, Texas, United States). The sequence plot of reported new cases of COVID-19, the total number of prescribed medications, BMD tests, and FRAX^®^ was plotted during the pandemic. Pearson's correlation coefficient was used to estimate the correlation between the number of prescribed medications, BMD tests, and FRAX^®^ and the reported cases of COVID-19 in the previous week.

### 3.3. Ethics

This study was approved by the research ethics committee of the Endocrinology and Metabolism Research Institute of Tehran University of Medical Sciences with the ID: IR.TUMS.EMRI.REC.1400.089. We utilized the pseudonymized data regarding the number of prescriptions for each evaluated testing and medication from IHIO. Therefore, no informed consent was obtained since no personal or confidential data of patients was used. Also, the authors did not have access to information that could identify individual participants during or after data collection. This research was carried out with the support of the Endocrinology and Metabolism Research Institute, Tehran University of Medical Sciences, Tehran, Iran, under project code 1400-02-103-1177.

## 4. Results

Concerning tests, a total number of 4,901,027 BMD tests and lab test prescriptions were evaluated. Among them, 62,718 prescribed BMD tests, 2,862,871 prescribed serum vitamin D tests, and 1,952,600 prescribed serum calcium tests were assessed. Regarding medications, 446,791 prescriptions composed of 388,519 alendronate and 58,272 calcitonin prescriptions were evaluated.

### 4.1. Osteoporosis Diagnosis by Bone Mineral Density Test, Fracture Risk Assessment Tool, and Laboratory Tests

Results of the Mann-Whitney test comparing median monthly usage of the osteoporosis diagnosis tests before and during the pandemic are presented in Table 1. 

**Table 1.** Results of the Mann-Whitney Test Comparing Median Monthly Usage of the Osteoporosis Diagnosis Tests Before and During the Pandemic

Tests and Pandemic Order	Number of Months	Median (Min-Max)	IQR	P-Value	Relative Change in Median (%)
**BMD test**				0.016	-63.1
Before	10	2151 (2036 - 2596)	174		
During	25	794 (134 - 5470)	1297		
**FRAX** ^**®**^^**a**^				0.362	+14.7
Before	11	719 (166 - 1001)	202		
During	26	824.5 (259 - 1447)	402		
**Serum vitamin D level tests**				< 0.001	+13134.4
Before	6	741.5 (32 - 1087)	359		
During	26	98133 (1148 - 233473)	69698		
**Serum calcium level tests**				< 0.001	+21273.5
Before	6	305.5 (11 - 400)	121		
During	26	65296 (438 - 146511)	49527		

Abbreviation: IQR, interquartile range; BMD, bone mineral density.

^a^ Regarding the Mann-Whitney test for FRAX^®^, data was assessed from March 2019 to March 2022

The results of the Mann-Whitney test showed a significant decrease in the median number of prescribed BMD tests per month during the pandemic compared to before it (P-value = 0.016). The analysis indicated a nonsignificant increase in FRAX^® ^usage after the beginning of the pandemic (P-value = 0.362). This was contrary to the significant increase in the median number of prescriptions per month for both laboratory tests after the beginning of the COVID-19 pandemic (P-value < 0.001) (Table 1). 

**Figure 1.**
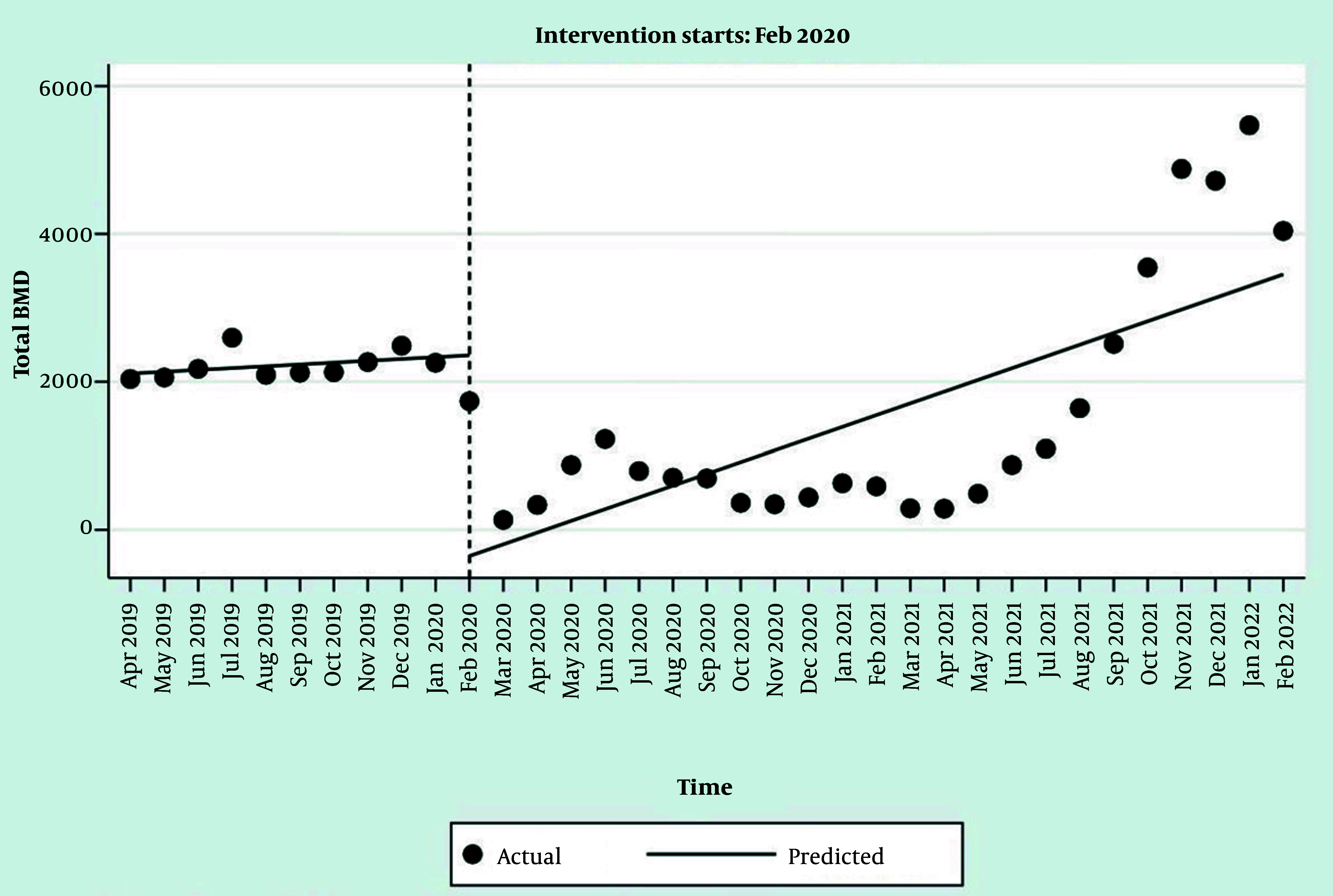
Interrupted time series regression analysis of bone mineral density (BMD) prescriptions

**Figure 2.**
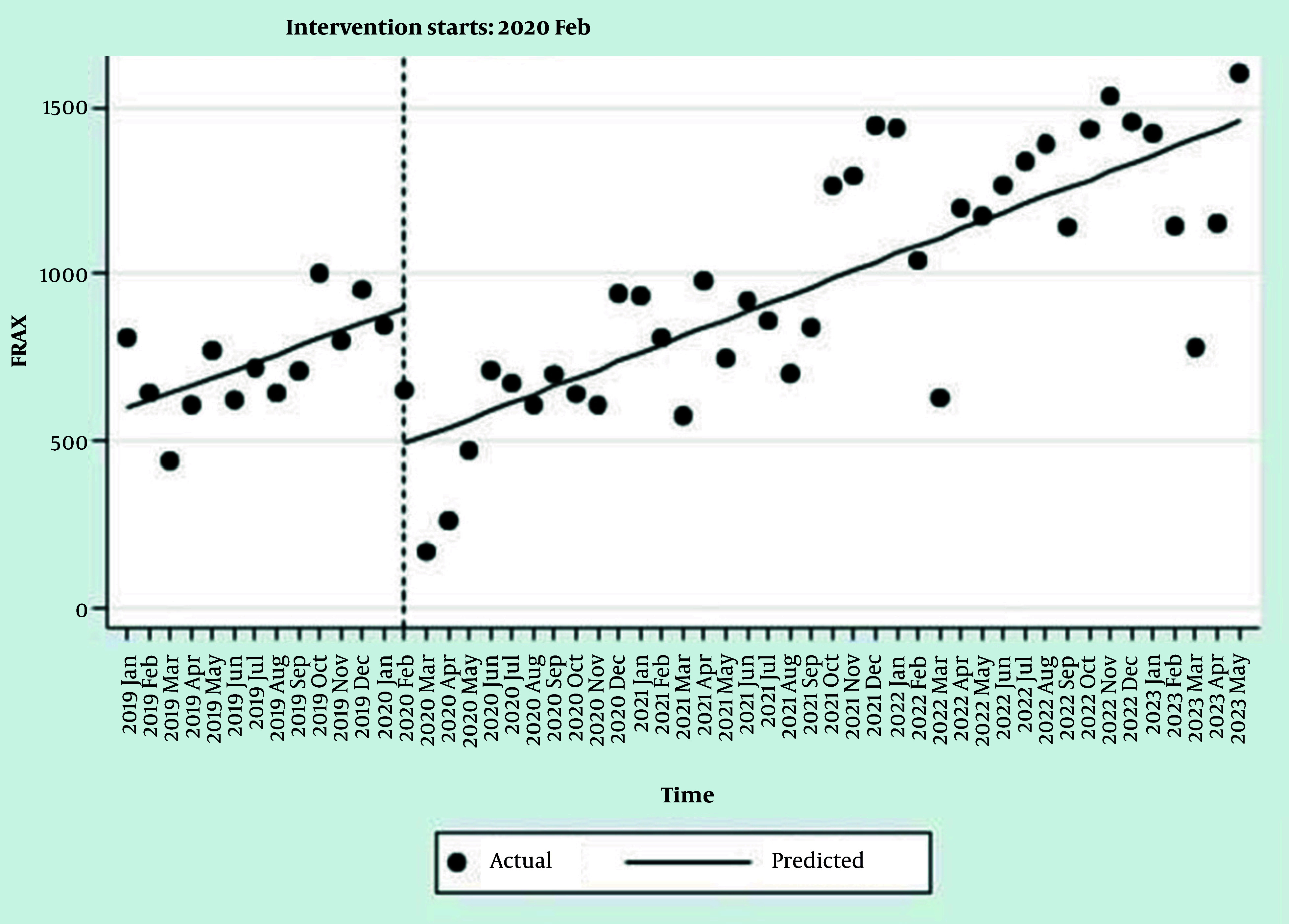
Interrupted time series regression analysis of FRAX^®^ usage

The interrupted time series regression results for the prescribed tests showed that, except for BMD tests, the trends in the number of laboratory tests and FRAX^® ^were significant before the COVID-19 pandemic (Table 2, Figures 1, and 2, and Appendices 1 and 2 in Supplementary File). With the onset of the pandemic in Iran in February 2020, the number of prescriptions for BMD tests and the FRAX^®^ usage decreased significantly (Table 2, Figures 1, and 2). The model estimates a baseline of 2,110.2 BMD test prescriptions at the start of the observation period.

**Table 2.** Results of Interrupted Time Series Regression on the Impact of the COVID-19 Pandemic on Prescriptions for Tests and FRAX^®^ Usage

Tests and Parameters	Regression Coefficient	95% Confidence Interval		P-Value
		Lower Limit	Upper Limit	
**BMD test**				
Intercept	2110.2	1928.6	2291.7	< 0.001
The trend before the pandemic	24.7	-1.1	50.5	0.060
Changing levels after the beginning of the pandemic	-2583.3	-3602.2	-1564.4	< 0.001
The trend after the beginning of the pandemic	142.5	51.7	233.3	0.003
Changing trends after the beginning of the pandemic compared to before the pandemic	117.8	25.2	210.4	0.014
**FRAX** ^**®**^				
Intercept	594.3	431.9	756.7	< 0.001
The trend before the pandemic	23.5	3.8	43.2	0.02
Changing levels after the beginning of the pandemic	-410.5	-596.3	-224.8	< 0.001
The trend after the beginning of the pandemic	24.8	16.9	32.8	< 0.001
Changing trends after the beginning of the pandemic compared to before the pandemic	1.4	-19.8	22.5	0.898
**Serum vitamin D level tests**				
Intercept	278.9	-6.8	564.6	0.055
The trend before the pandemic	180.8	105.8	255.7	< 0.001
Changing levels after the beginning of the pandemic	71579.4	2790.2	140368.7	0.042
The trend after the beginning of the pandemic	2959.9	-1.48e3	7404.8	0.18
Changing trends after the beginning of the pandemic compared to before the pandemic	2779.1	-1670.9	7229.2	0.211
**Serum calcium level tests**				
Intercept	127.5	-6.2	261.2	0.061
The trend before the pandemic	62.1	27.4	96.9	0.001
Changing levels after the beginning of the pandemic	814.1	-2334.8	3963.1	0.601
The trend after the beginning of the pandemic	876.3	-2.27e3	4022.8	0.572
Changing trends after the beginning of the pandemic compared to before the pandemic	127.5	-6.2	261.2	0.061

Abbreviation: BMD, bone mineral density.

Before the pandemic, the number of BMD test prescriptions was increasing by an average of 24.7 tests per month. At the onset of the pandemic, there was an immediate and statistically significant drop of approximately 2,583 BMD test prescriptions in a single month. After the pandemic began, the monthly trend in BMD test prescriptions became significantly steeper. The new trend was 117.8 units higher than the old trend, resulting in a new monthly increase of 142.5 prescriptions per month.

Despite this, the number of prescribed serum vitamin D tests increased statistically significantly, and a nonsignificant increase in the number of serum calcium test prescriptions was observed (Table 2 Appendices 1 and 2 in Supplementary File). The trend in the number of all tests and FRAX^®^ usage after the beginning of the COVID-19 pandemic (after the onset of the event) showed an increase, which was statistically significant only for BMD and FRAX^®^. At the onset of the pandemic, there was an immediate and statistically significant drop of approximately 411 instances of FRAX^®^ usage in a single month. After the pandemic, the monthly trend in FRAX^®^ usage did not change significantly (P = 0.898). The post-pandemic trend was nearly identical to the pre-pandemic trend, with a new monthly increase of 24.8 instances per month. The slope of the trends after the pandemic did not change significantly for most tests, except for BMD (Table 2, Figures 1, and 2, and Appendices 1 and 2 in Supplementary File).

### 4.2. Medications

An overview of osteoporosis medication prescriptions, including alendronate, calcitonin, and total medications, is presented in Table 3. These prescriptions were compared between 10 months before and 26 months during the pandemic. The results of the Mann-Whitney test showed that there was a significant decrease in the median number of prescriptions for alendronate, calcitonin, and total medications per month during the pandemic compared to before it (P-value < 0.001) (Table 3). 

**Table 3.** Results of the Mann-Whitney Test Comparing Median Monthly Prescriptions for Medications Before and During the Pandemic

Medications and Pandemic Order	Number of Months	Median	IQR	P-Value
**Alendronate**				< 0.001
Before	10	20587	836	
During	26	8303	6735	
**Calcitonin**				< 0.001
Before	10	3759.5	250	
During	26	692	1339	
**Total**				< 0.001
Before	10	24247.5	809	
During	26	8883.5	8135	

Abbreviation: IQR, interquartile range.

**Figure 3.**
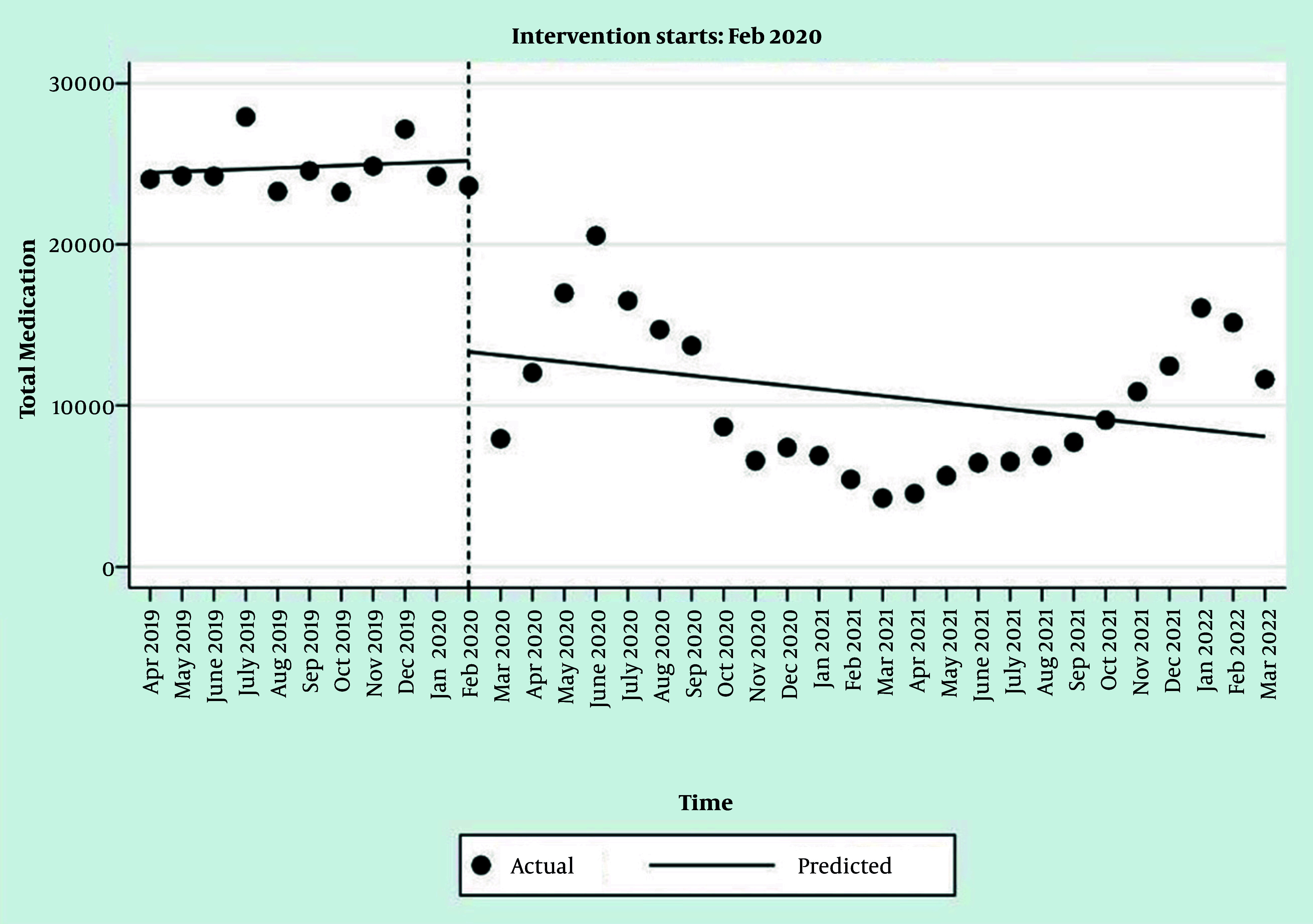
Interrupted time series regression analysis of total medication prescriptions

The interrupted time series regression results for the number of prescriptions for alendronate, calcitonin, and total medications are available in Table 4. Results showed that the trend of changes in the number of prescribed medicines before the COVID-19 pandemic was almost flat (P-value > 0.05) (Table 4, Figure 3, Appendices 3 and 4 in Supplementary File). Following the COVID-19 pandemic, the number of prescriptions for alendronate and calcitonin decreased statistically significantly (Table 4, Appendices 3 and 4 in Supplementary File). The trend of changes in the number of these prescriptions after the beginning of the pandemic was also approximately flat (P-value > 0.05) (Table 4, Figure 3, Appendices 3, 4 in Supplementary File). At the onset of the pandemic, there was an immediate and statistically significant drop of approximately 9,592 alendronate prescriptions in a single month. The change in trend after the pandemic was not statistically significant (P = 0.086), indicating the post-pandemic trend remained approximately flat. The pre-pandemic trend for calcitonin prescriptions was flat, with no statistically significant change per month (P = 0.134). Similar to alendronate, there was an immediate and statistically significant drop of approximately 2,272 calcitonin prescriptions in a single month at the onset of the pandemic.

**Table 4.** Results of Interrupted Time Series Regression on the Impact of the COVID-19 Pandemic on Prescriptions for Medications

Medications and Parameters	Regression Coefficient	95% Confidence Interval		P-Value
		Lower Limit	Upper Limit	
**Alendronate**				
Intercept	20564.2	19578.9	21549.5	< 0.001
The trend before the pandemic	99.8	-89.7	289.4	0.291
Changing levels after the beginning of the pandemic	-9592.0	-14217.6	-4966.4	< 0.001
The trend after the beginning of the pandemic	-184.4	-475.3	106.5	0.206
Changing trends after the beginning of the pandemic compared to before the pandemic	-284.3	-611.0	42.5	0.086
**Calcitonin**				
Intercept	3880.1	3662.5	4097.8	< 0.001
The trend before the pandemic	-24.8	-57.6	8.0	0.134
Changing levels after the beginning of the pandemic	-2272.3	-3215.2	-1329.5	< 0.001
The trend after the beginning of the pandemic	-26.0	-93.2	41.2	0.436
Changing trends after the beginning of the pandemic compared to before the pandemic	-1.2	-72.5	70.1	0.973
**Total**				
Intercept	24444.3	23249.5	25639.2	< 0.001
The trend before the pandemic	75.0	-143.7	293.8	0.490
Changing levels after the beginning of the pandemic	-11864.4	-17380.8	-6347.9	<0.001
The trend after the beginning of the pandemic	-210.4	-566.1	145.2	0.237
Changing trends after the beginning of the pandemic compared to before the pandemic	-285.5	-678.7	107.8	0.149

**Figure 4.**
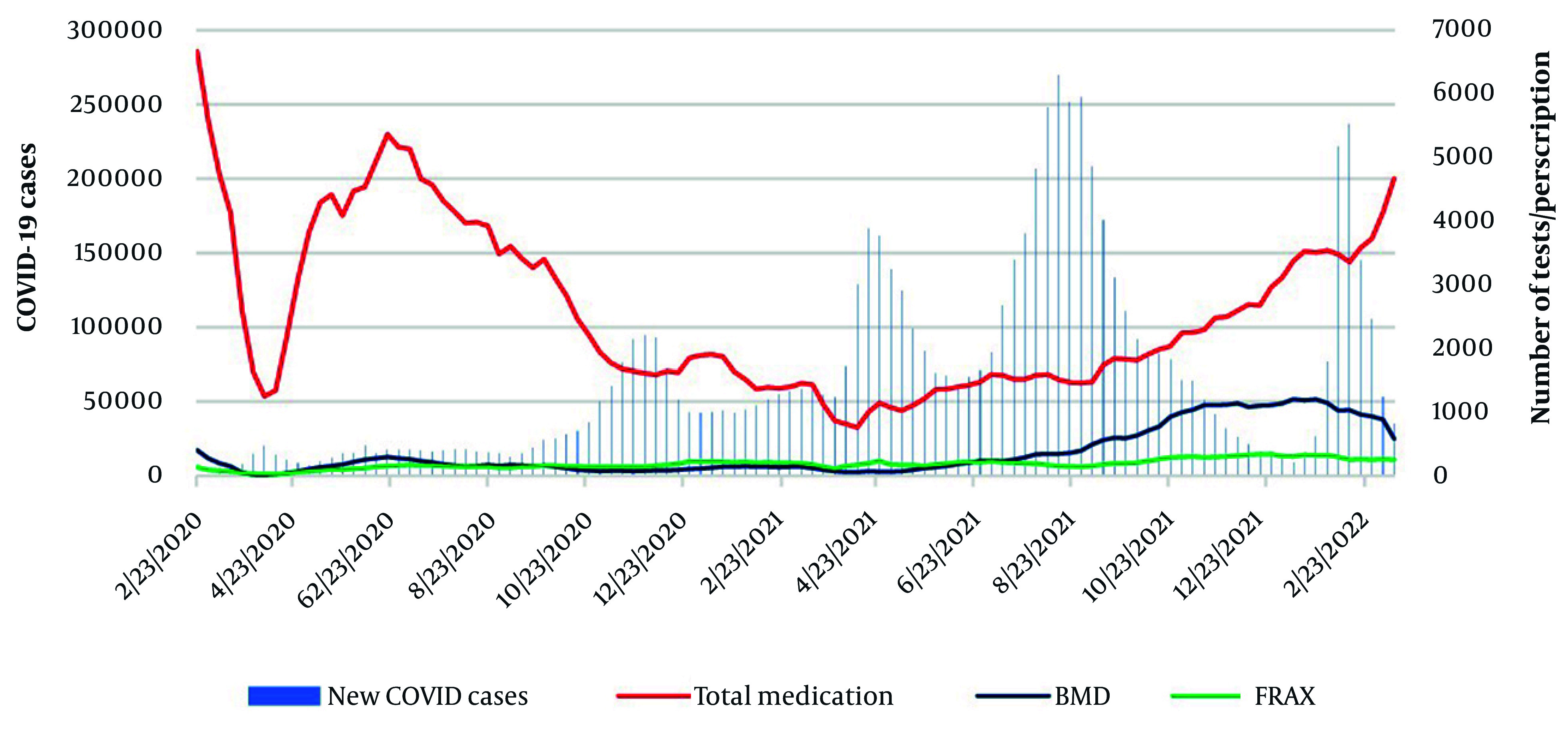
Sequence plot of reported new COVID-19 cases, total medication prescriptions, bone mineral density (BMD) tests, and FRAX^®^ usage during the COVID-19 pandemic

Figure 4 presents the sequence plot of the number of reported new COVID-19 cases in Iran, the number of prescriptions for alendronate and calcitonin, BMD tests, and FRAX^®^ usage during the COVID-19 pandemic. The correlation between the number of prescriptions and new COVID-19 cases in the prior week was -0.402 (< 0.001). The correlation of the number of prescribed alendronate and calcitonin with COVID-19 cases was -0.358 (< 0.001).

The findings from cross-correlation analysis showed no strong correlation between the surges in COVID-19 cases and the rates of prescriptions for osteoporosis diagnosis and treatment (Appendix 5 in Supplementary File).

## 5. Discussion

This cross-sectional study using IHIO data compared osteoporosis diagnosis and treatment before and during the COVID-19 pandemic. Regarding diagnosis, the number of prescriptions for BMD tests, serum vitamin D tests, serum calcium tests, and the number of FRAX^®^ usage with the IP of Iran were assessed. Results of the interrupted time series analysis as a more advanced statistical analysis method demonstrated a statistically significant decrease in the prescriptions for BMD test and FRAX^®^ usage and a significant increase in prescriptions for serum vitamin D test. Furthermore, a nonsignificant increase in serum calcium tests was demonstrated. Concerning treatment, the number of prescriptions for alendronate and calcitonin was assessed. Evaluations represented a statistically significant decrease in the prescriptions for alendronate and calcitonin during the pandemic compared to before its beginning.

According to statistical analysis, the results of this investigation demonstrated a significant decrease in the number of prescriptions for BMD tests after the pandemic initiation. This was in line with a survey conducted among healthcare workers affiliated with the International Osteoporosis Foundation to evaluate their practice regarding osteoporosis management during the COVID-19 pandemic. The study reported that 29% of healthcare workers accelerated the arrangement of DXA scans while 11% relied on clinical risk assessment tools, such as FRAX^®^, 29% evaluated patients based on clinical risk assessment tools with DXA planned for future, 33% scheduled DXA for a time when the transmission risk of COVID-19 decreased, and 5% reported either closed DXA units or referring patients to a specific clinic service for osteoporotic fractures. The survey data raised concerns regarding the lack of adherence to the conventional gold standard assessment for osteoporosis patients during the pandemic (12).

As presented in another study, a significant decrease was noted in BMD test numbers during the COVID-19 pandemic from March to June 2020. In contrast to other healthcare resource utilizations, BMD test numbers remained 25% lower during the COVID-19 period from June to September 2020 compared to the average numbers during the control period (7). Based on a systematic review conducted in the United Kingdom (UK) during the COVID-19 pandemic, BMD tests, primarily provided by secondary-care facilities in the UK, have been compromised and disrupted. Diagnostic imaging and radiological procedures have been prioritized based on clinical urgency, leading to deprioritization or temporary breaks in DXA services in many centers. Stringent infection control measures and social distancing have decreased throughput in centers where DXA services have continued to operate. Frail and elderly patients, who are at higher risk for fragility fractures related to osteoporosis, have been hesitant to attend hospitals due to fears of virus transmission. As a result, there was a 73% decrease in the number of performed DXA scans in June 2020 compared to the previous year, leading to a backlog that may take months to clear and return waiting lists to acceptable levels in the UK (8). Also, a lack of DXA measurements and higher rates of appointment cancellation were observed in the Netherlands (13).

Regarding assessing the number of FRAX^®^ with the IP address of Iran, a significant decrease was reported by the interrupted time series. The results of our study were consistent with the results of a descriptive study that indicated a substantial reduction in global usage of FRAX^®^, averaging 58% and peaking at 96%. Based on this international report, two-thirds of the 66 evaluated countries and territories showed a decrease of at least 50% (14).

Our study demonstrated an increase in the number of prescriptions for serum vitamin D tests, which aligns with the results of an investigation conducted in Canada. In the mentioned study, the number of Vitamin D lab tests exhibited a modest decline from the March to June 2020 COVID-19 period, followed by a significant increase in the subsequent June to September 2020 COVID-19 period (7). As stated by previous research findings, in patients admitted to the hospital with COVID-19 infection, there was an observed correlation between lower levels of serum vitamin D levels and the severity of the disease, as well as an increased likelihood of requiring admission to the intensive care unit and experiencing higher mortality rates (15). Hence, this increase in prescriptions for serum vitamin D tests can be consistent with the correlation between serum vitamin D levels and COVID-19 severity.

Furthermore, during the COVID-19 pandemic, disorders of calcium metabolism, specifically hypocalcemia, were reported in patients diagnosed with COVID-19 (16). Hypocalcemia was reported as a prevalent biochemical finding among hospitalized patients. Its prevalence was reported to be approximately between 62.6% to 87.2%. Hypocalcemia was demonstrated to be efficient in predicting mortality as well as prognosis and resulted in complications (17, 18). Our results showed a rise in the prescriptions for serum calcium tests, although this increment was not statistically significant in the interrupted time series test. Since some prescriptions are related to inpatient cases, an increase in serum calcium assessment during the COVID-19 pandemic has been observed. This increment can be supported by the previous study, which assessed the role of serum calcium imbalance in the prognosis of COVID-19 severity among hospitalized patients (19). According to the aforementioned research findings, an association between low serum calcium levels and the severity of the disease was observed.

The management gap of osteoporosis was a healthcare challenge exacerbated by the beginning of the COVID-19 pandemic. A substantial treatment deficit on a global scale in the management of osteoporosis exists, with only approximately 20% of patients receiving adequate treatment following a hip fracture, the critical period with the highest susceptibility to subsequent fractures (8). As demonstrated by the findings of another study, in a population suffering from post-fracture conditions, the assessment and treatment of bone loss are frequently overlooked (19). In light of previous research, despite significant advancements in osteoporosis treatment, a considerable number of high-risk patients continue to remain untreated for fractures, either due to lack of medication prescription or non-adherence to prescriptions (20). This worsening of the treatment gap resulted from perceiving the management of osteoporosis as a low priority in clinical settings after the pandemic (8).

Our study demonstrated a disruption in prescribing for alendronate after the beginning of the COVID-19 pandemic. This finding supports the results of a study conducted in Canada, which reported a reduction in rates of treatment initiation with oral bisphosphonates by 43%. Based on this study, from June to September 2020, the initiation of bisphosphonate treatments was reduced by 14% compared to the weighted average control time (7). A study conducted in the Netherlands found that ongoing treatment and follow-up for osteoporotic patients remained feasible despite delays caused by the pandemic. However, this situation was less favorable for initiating treatment, which experienced delays or, in some cases, was not initiated. Also, many patients with increased Garvan scores or FRAX^®^ remained untreated. Furthermore, considerable challenges were observed in addressing fracture prevention during the COVID-19 pandemic (13). These results are supported by the outcomes of our study, which observed a significant decrease in the prescriptions for osteoporosis medications.

The present study had some limitations. One was that the data on prescriptions for other osteoporosis medications and diagnostic tests were unavailable. Moreover, there was a lack of access to the data on prescriptions for people whose costs are not covered by the IHIO, including people not benefiting from any health insurance services or benefiting from other health insurance organizations in Iran. Another limitation was the confined data access. For instance, despite other variables, the data on the numbers of serum vitamin D tests and serum calcium tests were unavailable for more than six months before the pandemic, and the data on the number of prescriptions for serum parathyroid hormone levels tests and serum phosphorus levels tests were not accessible. Furthermore, the data on prescriptions for medications whose costs are not covered by IHIO was inaccessible.

The external validity of our results should be interpreted in light of the data source and study design. We analyzed prescription-level data from the IHIO, which covers a substantial proportion of the population but not all healthcare payers in Iran; therefore, our findings principally reflect prescribing patterns within this insured population and the national health system during 2019–2022. Differences in healthcare structure, reimbursement policies, access to diagnostic services, and clinician behaviour in other countries may limit direct transferability. Nonetheless, the large sample size, national scope of the IHIO dataset, and the real-world nature of the data strengthen the applicability of the observed temporal trends to similar middle-income settings.

### 5.1. Conclusions

In conclusion, the results of this study demonstrated a significant decrease in the number of prescriptions for BMD tests and FRAX^®^ usage in Iran, a significant increase in prescriptions for serum vitamin D tests, and a nonsignificant increase in prescriptions concerning serum calcium tests during the COVID-19 pandemic, likely due to their association with COVID-19 management. These results indicated a disruption in osteoporosis diagnosis during the COVID-19 pandemic. It also illustrated significantly lower rates of prescriptions for medications during the pandemic, indicating an interruption in osteoporosis treatment amidst the COVID-19 pandemic. These findings highlight the remarkable need for developing more structured facilities for managing NCDs like osteoporosis, which would help avoid the mismanagement of such diseases amidst public health crises like the COVID-19 pandemic. However, it had some limitations, and further studies are needed to assess all aspects of this impact and to improve osteoporosis management in future health crises.

ijem-23-4-165816-s001.pdf

## Data Availability

Data will be available upon request from the corresponding author.
