# Supplementary material for: The Impact of the COVID-19 Pandemic on Osteoporosis Diagnosis and Treatment in Iran: A National Study
Source: Int J Endocrinol Metab. 2025 Oct 31;23(4):e165816. doi: 10.5812/ijem-165816 (PMC12915347; doi:10.5812/ijem-165816)
Supplement: ijem-23-4-165816-s001.pdf [file ijem-23-4-165816-s001.pdf]

“Supplementary Figures”

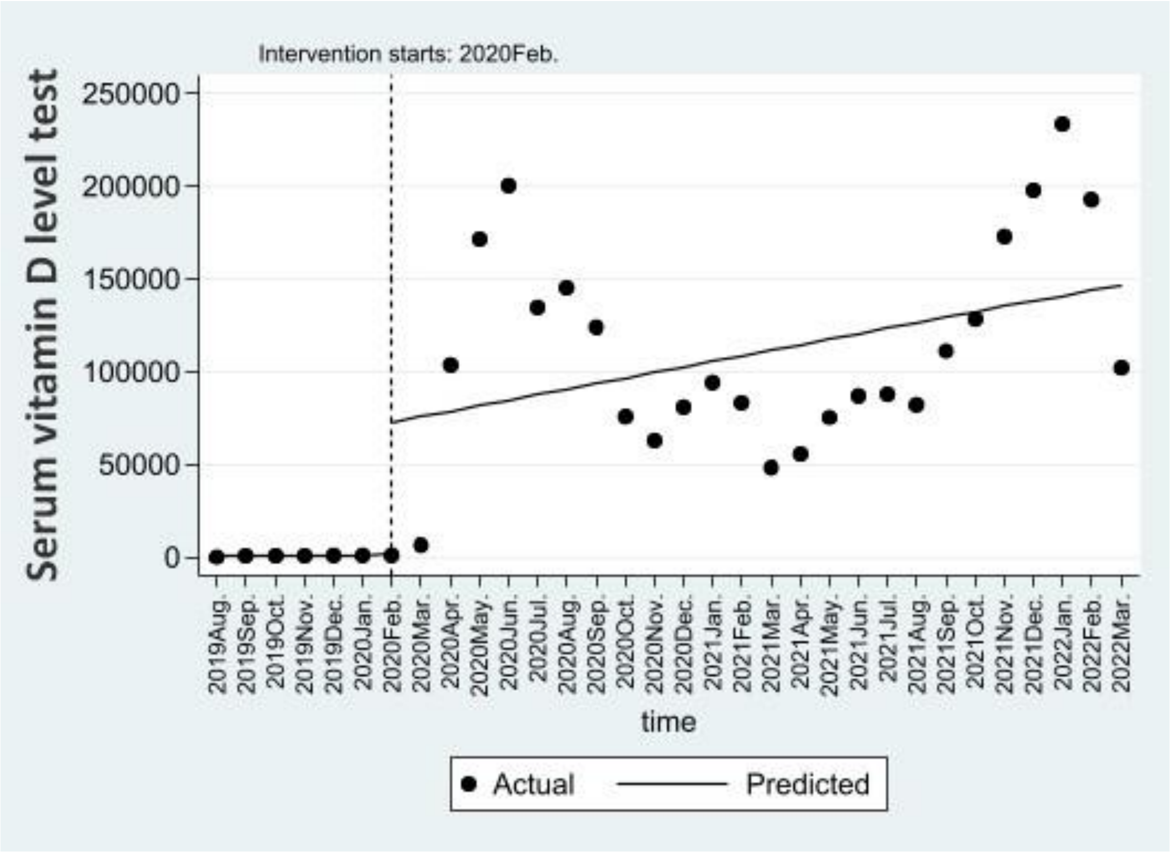

Appendix 1. Interrupted time series regression analysis of serum vitamin D test prescriptions

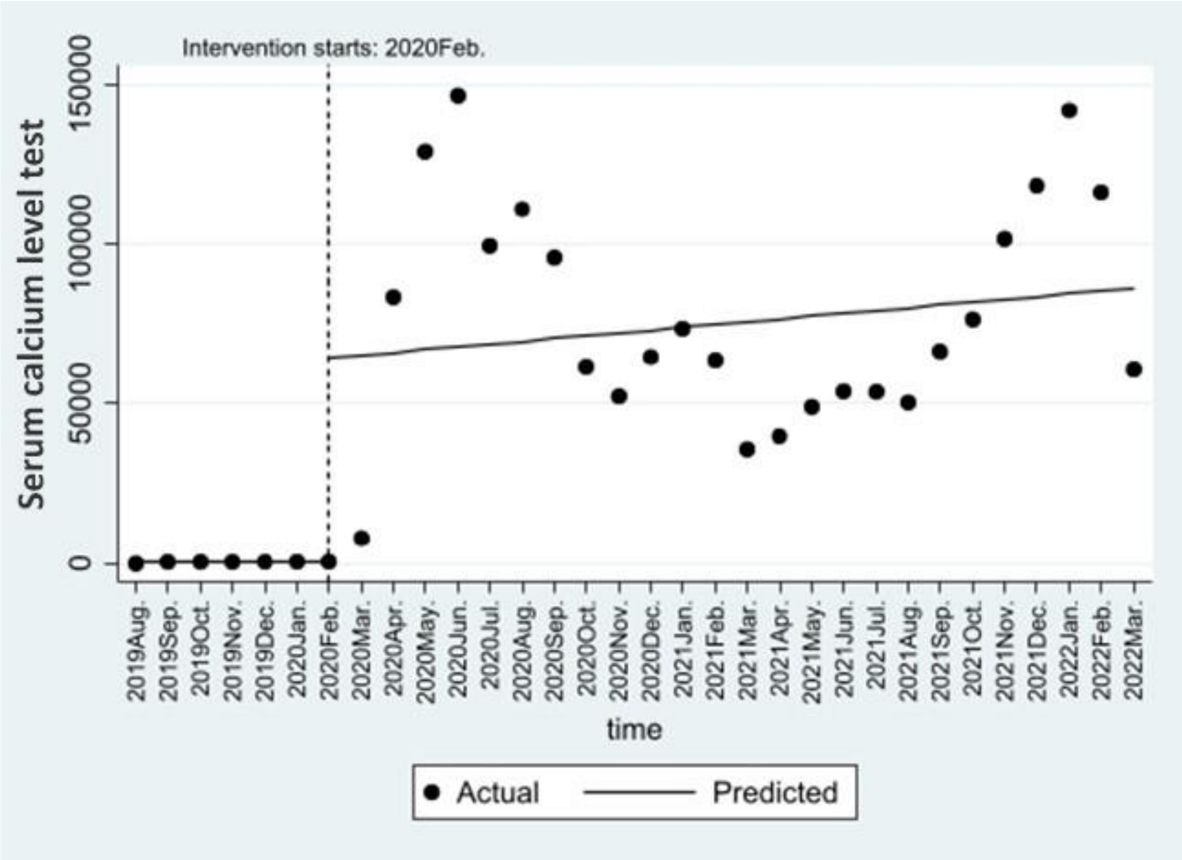

Appendix 2. Interrupted time series regression analysis of serum calcium test prescriptions

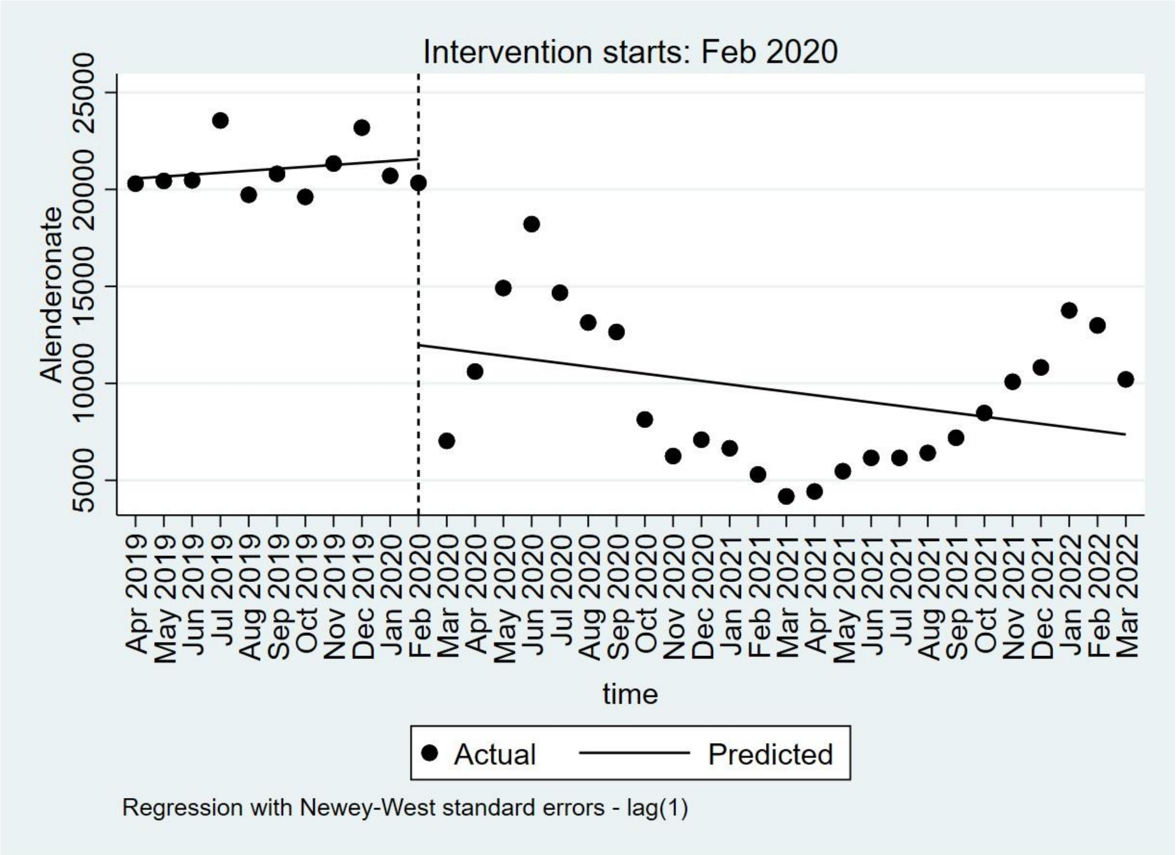

Appendix 3. Interrupted time series regression analysis of alendronate prescriptions

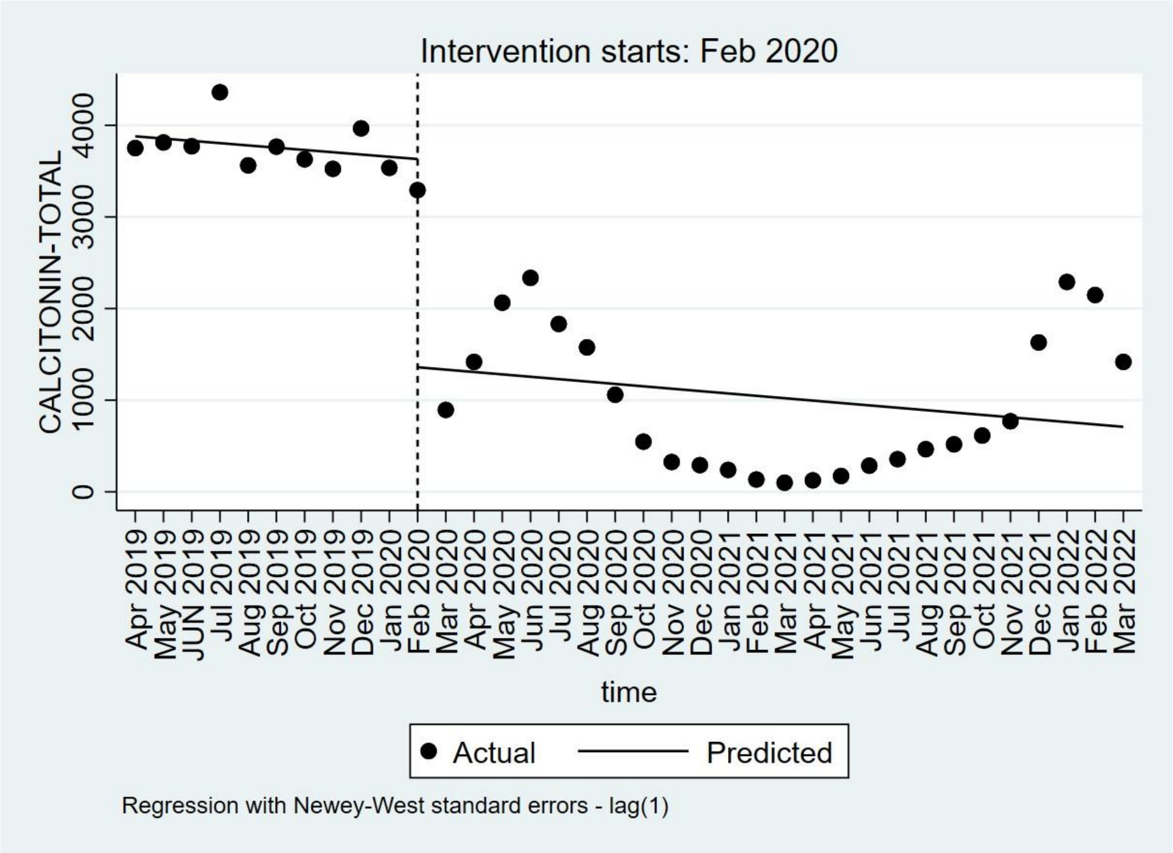

Appendix 4. Interrupted time series regression analysis of calcitonin prescriptions

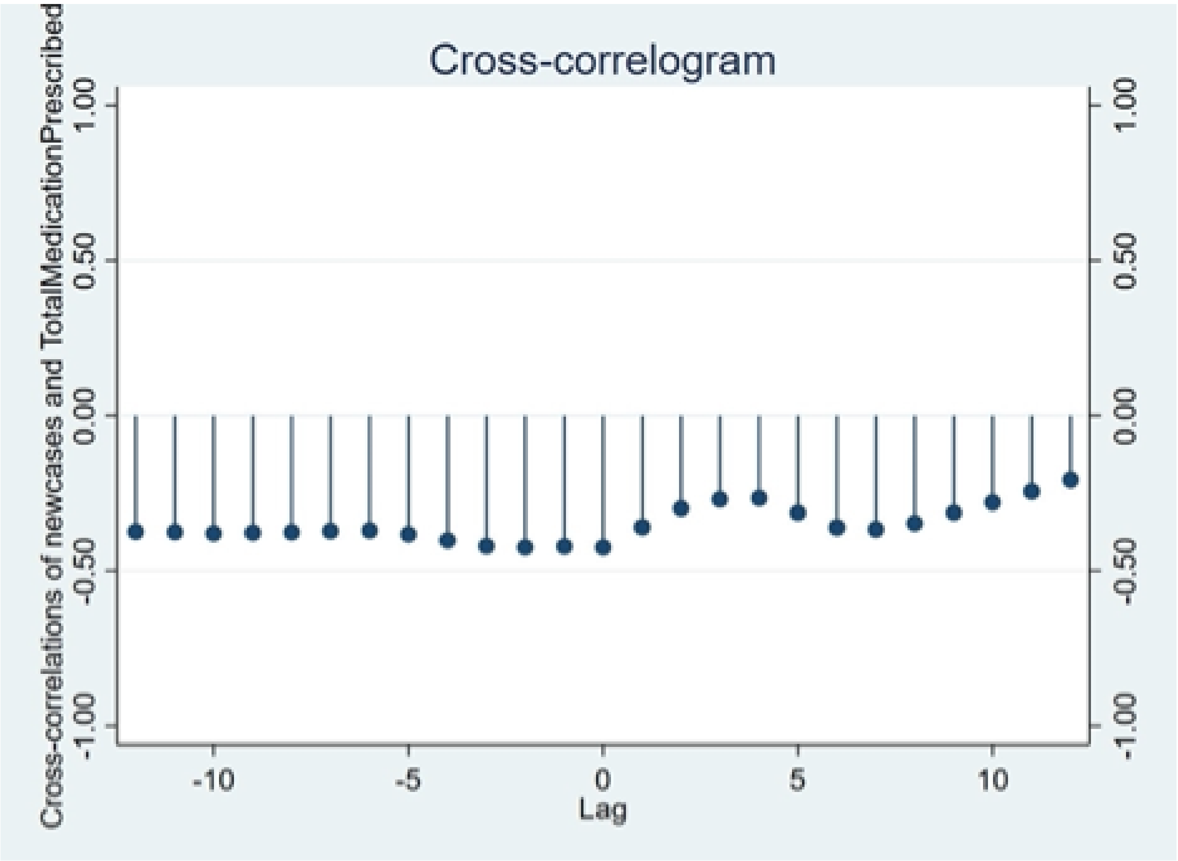

Appendix 5. Cross-correlation analysis of the correlation between the surges in COVID-19 cases and the rates of prescriptions for osteoporosis diagnosis and treatment
